# Supplementary material for: Odd skipped-related 1 identifies a population of embryonic fibro-adipogenic progenitors regulating myogenesis during limb development
Source: Nat Commun. 2017 Oct 31;8:1218. doi: 10.1038/s41467-017-01120-3 (PMC5662571; doi:10.1038/s41467-017-01120-3)
Supplement: Supplementary file 1 — Supplementary Information [file 41467_2017_1120_MOESM1_ESM.pdf]

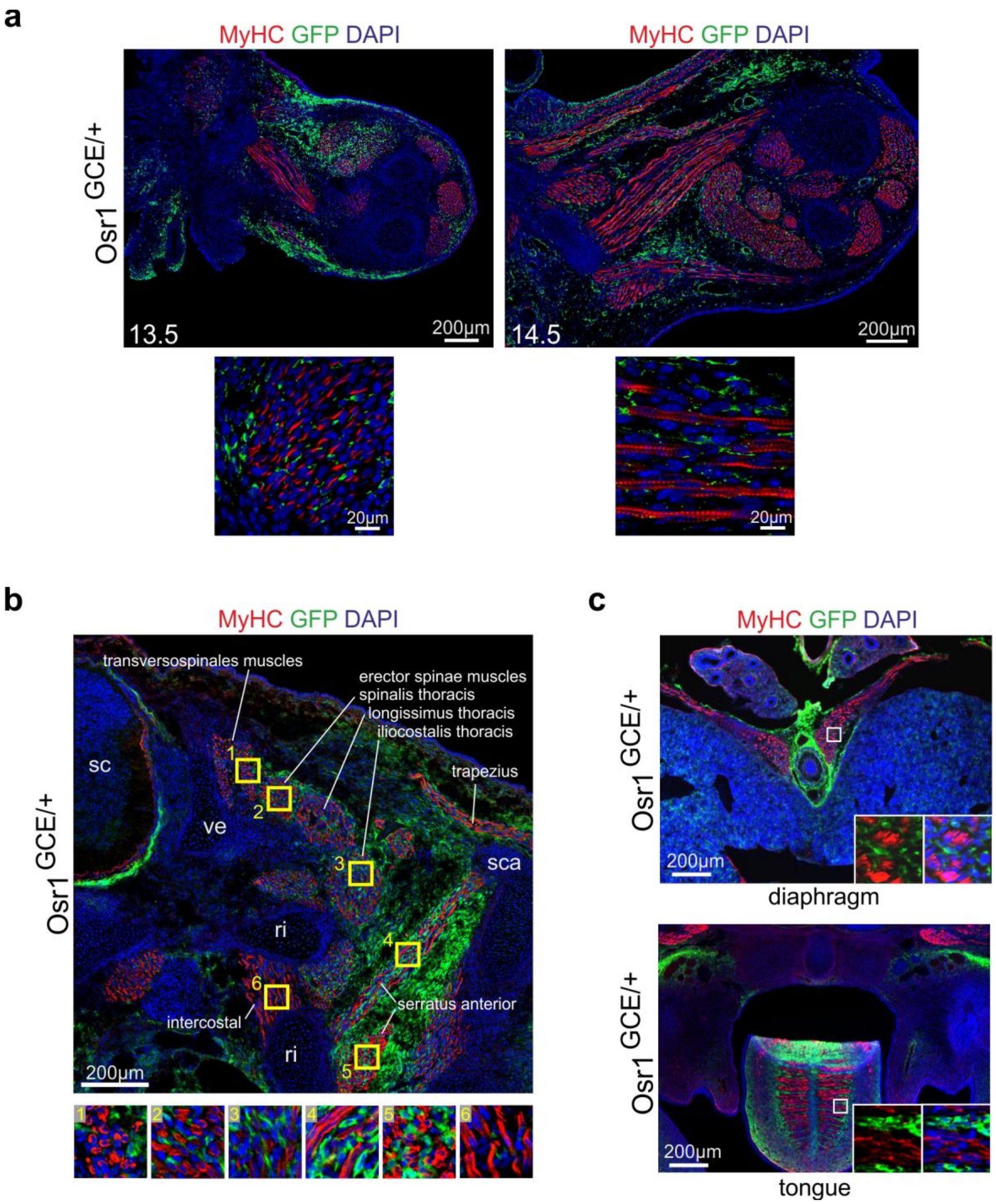

Supplementary Figure 1. Expression of *Osr1* analysed by immunolabelling for GFP expressed from the *Osr1^{GCE}* allele. (**a**) Overview of Osr1 expression pattern in E13.5 and E14.5 mouse hindlimbs. *Osr1* is expressed in irregular connective tissue cells in distinct regions of the limb. *Osr1* expression is associated with muscles, but also in the dermis. *Osr1* is expressed in cells interstitial to myofibers. Note that *Osr1* is expressed associated with some but not all muscles. (**b**) Osr1 expressing cells are found in other muscle groups as muscles of the shoulder girdle and superficial and deep back muscles or (**c**) diaphragm and tongue. Boxed regions in (b) are shown in the numbered panels below and are magnifications of the area. Muscle names are indicated in the overview picture. Ve: vertebral condensation, ri: rib condensation, Sca: scapula, sc: spinal cord. Images are representatives of n=3 experiments.

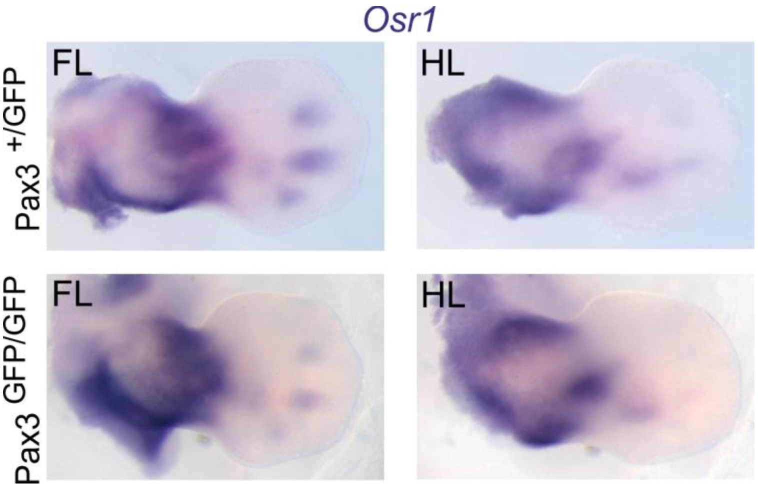

Supplementary Figure 2. Normal *Osr1* expression in muscleless limbs. *Osr1* expression assessed by whole mount *in-situ* hybridization on E12.5 limbs from *Pax3* heterozygous (control) or null (muscleless) embryos. Note that the expression pattern of *Osr1* is similar in both situations.

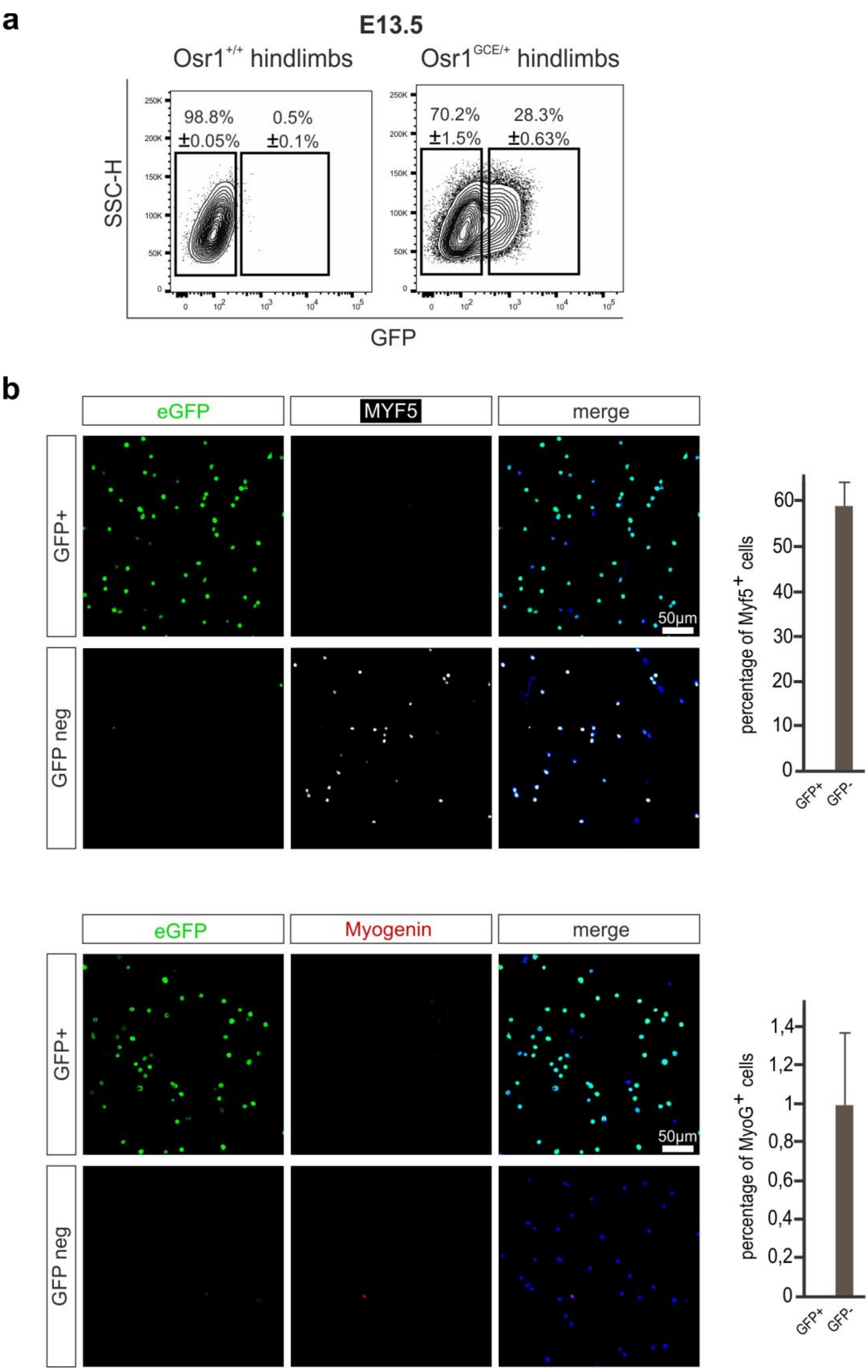

Supplementary Figure 3. Osr1 is not expressed in myogenic cells. (**a**) GFP$^+$ and GFP$^-$ cells were FACS isolated from E13.5 Osr1$^{GCE/+}$ fore- and hindlimbs. (**b**) FACS-isolated cells were cytospun and immunolabeled for Myf5 and Myogenin. No GFP expression was observed in myogenic cells, no Myf5$^+$ or Myogenin$^+$ cells were detected in the GFP$^+$ population. Quantification shown right (n=3). Myogenin expression was only sparsely detected in the GFP$^-$ population (approx. 1% of cells).

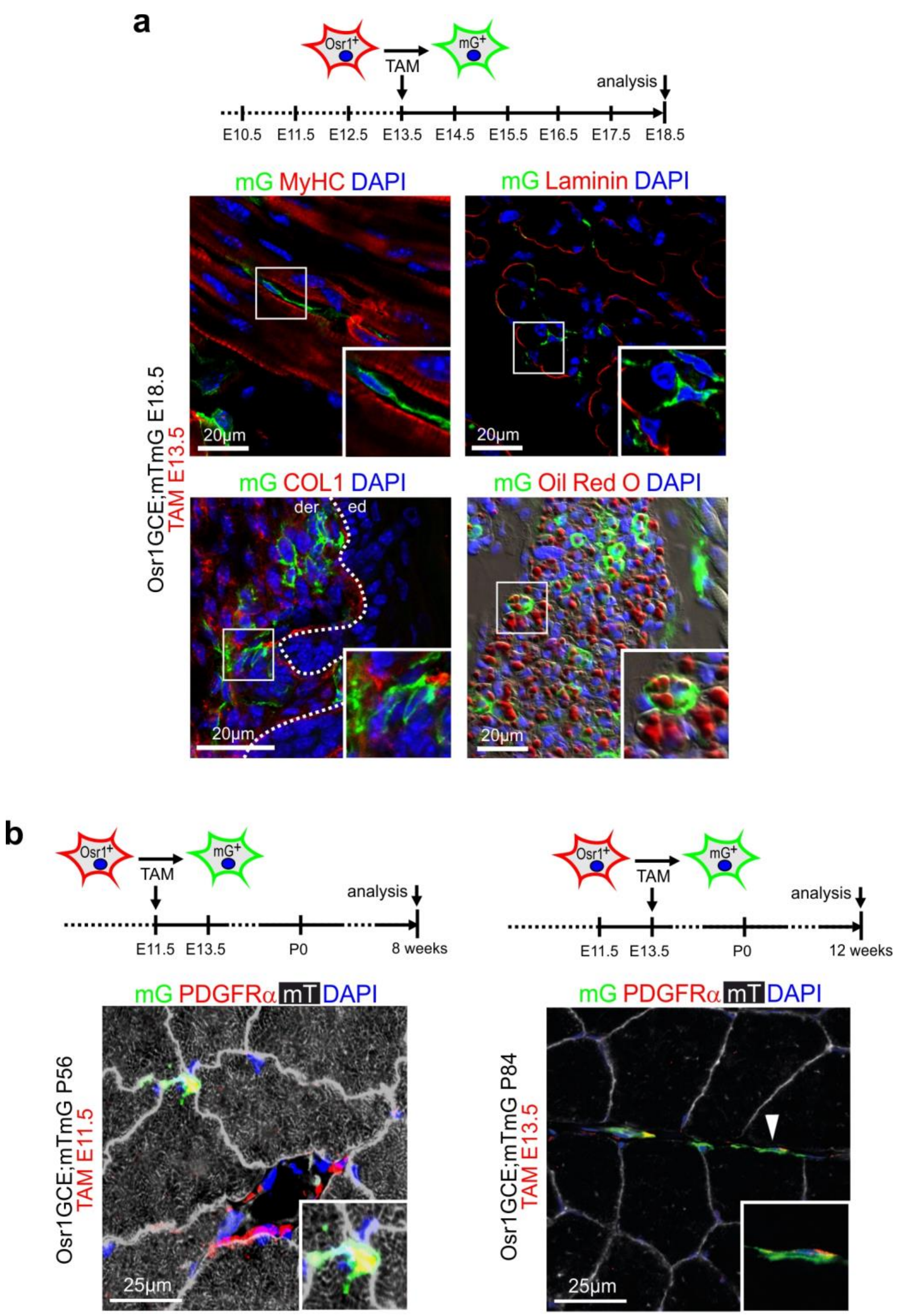

Supplementary Figure 4. In vivo differentiation potential of Osr1⁺ cells. (**a**) Tamoxifen induction of *Osr1^GCE/+^;R26R^mTmG/+^* mice was done at E13.5, analysis was performed at E18.5. Osr1⁺ cell descendants are found in the muscle interstitium (myofibers are labelled for MyHC or Laminin), in dermal fibroblasts embedded in a Collagen I (COL1) rich matrix and in adipose tissue (oil red O staining). Representative images of n=3 experiments are shown. (**b**) Tamoxifen induction of *Osr1^GCE/+^;R26R^mTmG/+^* mice was done at E11.5 or E13.5, analysis was performed at P56 (8 weeks) or P84 (12 weeks). Osr1 cell progeny (mG positive) cells are visible in the muscle interstitium, the cells coexpress PDGFRα. Representative images of n=3 experiments are shown.

a

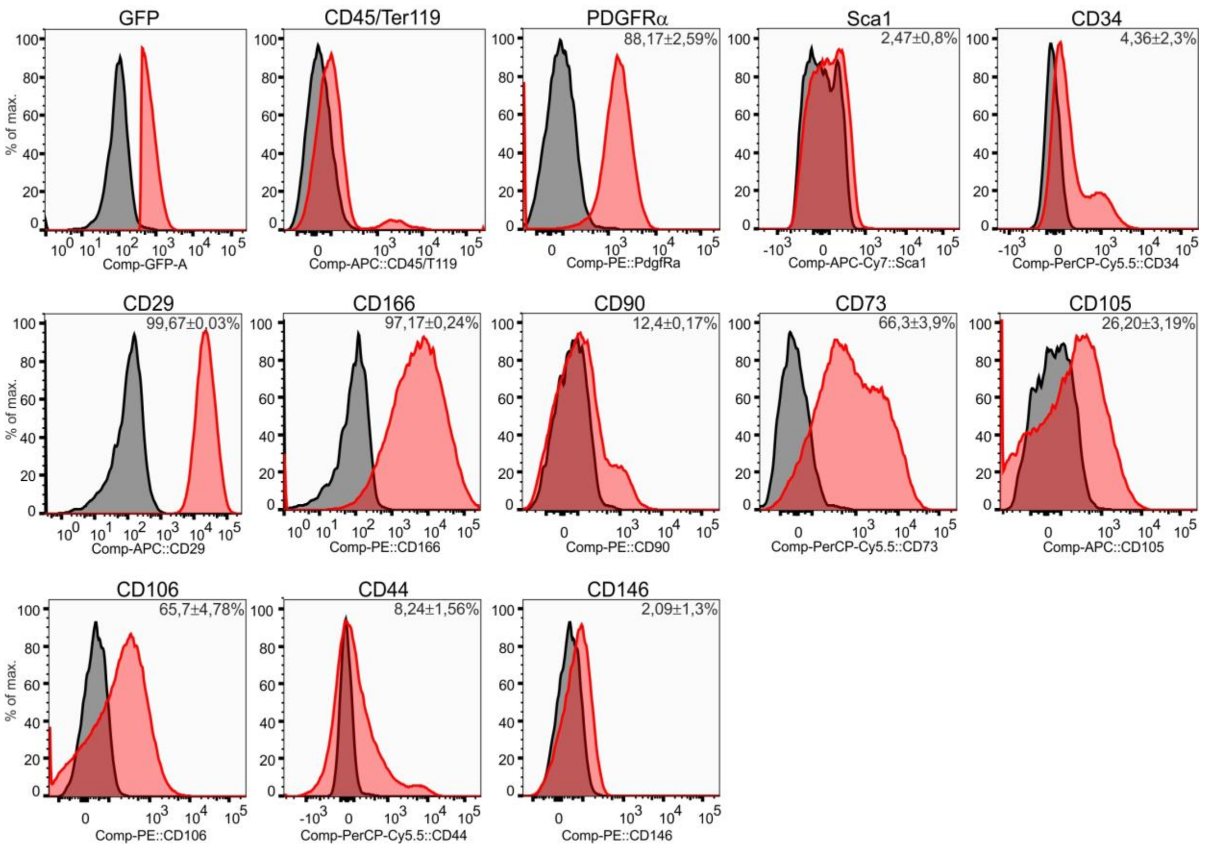

b

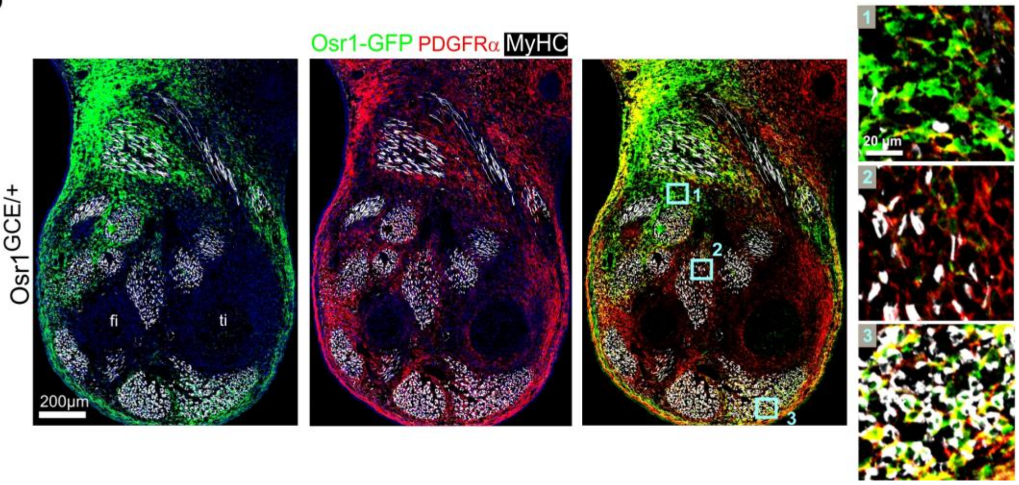

Supplementary Figure 5. Identity of E13.5 Osr1⁺ cells. (**a**) Osr1⁺ cells were sorted based on GFP expression; cells were negative for hematopoietic markers CD45 and Ter119. Osr1⁺ cells are positive for the FAP marker PDGFRα, but negative for adult FAP markers CD34 and Sca1 (Ly6A/E). Osr1⁺ cells are positive for FAP/MSC overlapping markers CD29 and CD166 and negative for CD90. Osr1⁺ cells are positive for MSC markers CD73, CD105, CD106 and negative for CD44 and CD146. Percentage values of positive cells are indicated, error represents s.e.m. (n=3). (**b**) Immunolabeling for Osr1-GFP and PDGFRα on sections of E13.5 $Osr1^{GCE/+}$ hindlimbs. Boxed regions are shown in the numbered panels to the right and are magnifications of the area. The expression patterns of Osr1-GFP and PDGFRα are largely overlapping thus confirming the FACS analysis, however regional PDGFRα expression in $Osr1^{-}$ cells can be seen. Ti: tibia; fi: fibula.

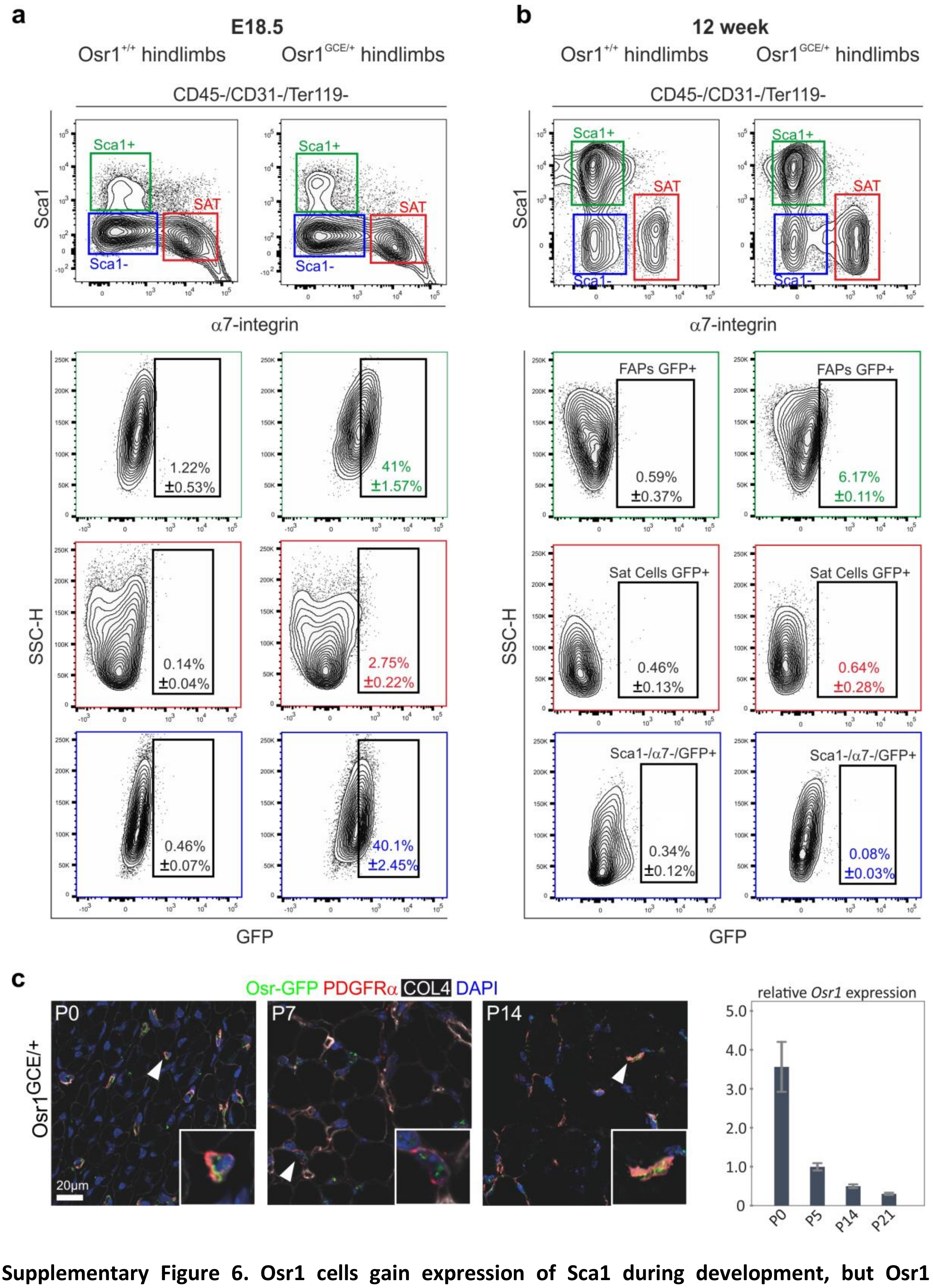

Supplementary Figure 6. Osr1 cells gain expression of Sca1 during development, but Osr1 is downregulated in FAPs after birth. (**a**) comparative FACS analysis of $Sca1^{+}$ cells from E18.5 and (**b**) from adult (12 week old) mouse limb muscles. Note that at E18.5 the majority of limb muscle interstitial cells are still $Sca1^{-}$ (blue square), but a $Sca1^{+}$ population is emerging (green square). In both populations Osr1-GFP^{+} cells are found. In adult $Sca1^{+}$ FAPs, only low GFP expression can be observed, indicating low Osr1 expression levels. Percentage values are indicated, error represents s.e.m. (n=3). (**c**) Immunolabeling for GFP on sections of $Osr1^{GCE/+}$ hindlimbs at the indicated time points after birth. Osr1 expression apparently decreases in the first weeks after birth, which is confirmed by RT-qPCR for *Osr1* on RNA from whole tibialis anterior muscle lysates of C57Bl6 wild type mice (right). Error bars indicate standard deviation.

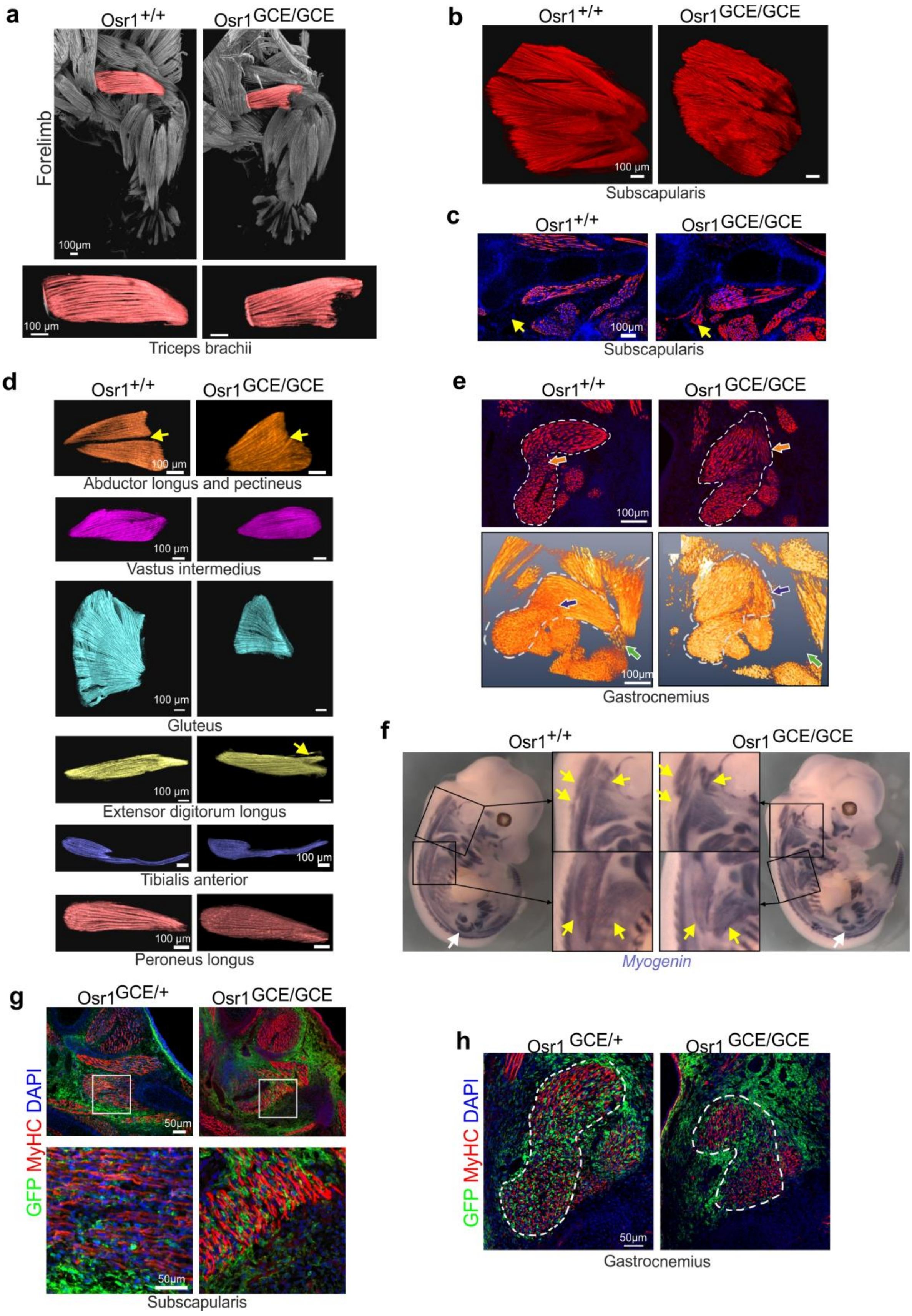

Supplementary Figure 7. Further examples of muscle patterning defects and presence of Osr1⁺ cells in *Osr1* mutant embryos. The forelimbs were less affected than the hindlimbs. Altogether similar defects were seen as in the hindlimbs, as (**a**) distal truncation as in the Triceps brachii, (**b**) disarrangement of myofibers shown in the subscapularis muscle and (**c**) appearance of ectopic fibers proximal to the subscapularis (arrows). (**d**) Further examples of mispatterning in the hindlimb: a defect in splitting of specific muscles like the Abductor longus and Pectineus muscles was observed. The

vastus intermedius muscle and the Gluteus maximus (pelvic girdle) muscle were altered in shape and size. In addition we observed ectopic myofibers at the distal tip of the Extensor digitorum longus (EDL) muscle, which was however overall unaffected, like the tibialis anterior and peroneus longus muscles. (**e**) The Gastrocnemius muscle is shown as 3D reconstruction (Amira) from individual, MyHC immunolabelled sections, alteration of shape and trajectory is visible. (**f**) Whole-mount ISH for Myogenin on E13.5 $Osr1^{+/+}$ and $Osr1^{GCE/GCE}$ embryos shows patterning defects of back muscles and shoulder girdle muscles (yellow arrows in magnification inserts) and the pelvic girdle muscles (white arrows). (**g, h**) *Osr1*-expressing cells are present in *Osr1* mutants at E13.5. *Osr1* gene activity traced by GFP expression was analysed in $Osr1^{GCE/+}$ and $Osr1^{GCE/GCE}$ embryos in the shoulder region (f) and the Gastrocnemius muscle (g) of the hindlimb. A similar pattern of muscle-associated and interstitial GFP^{+} cells was observed in $Osr1^{GCE/+}$ and $Osr1^{GCE/GCE}$ embryos.

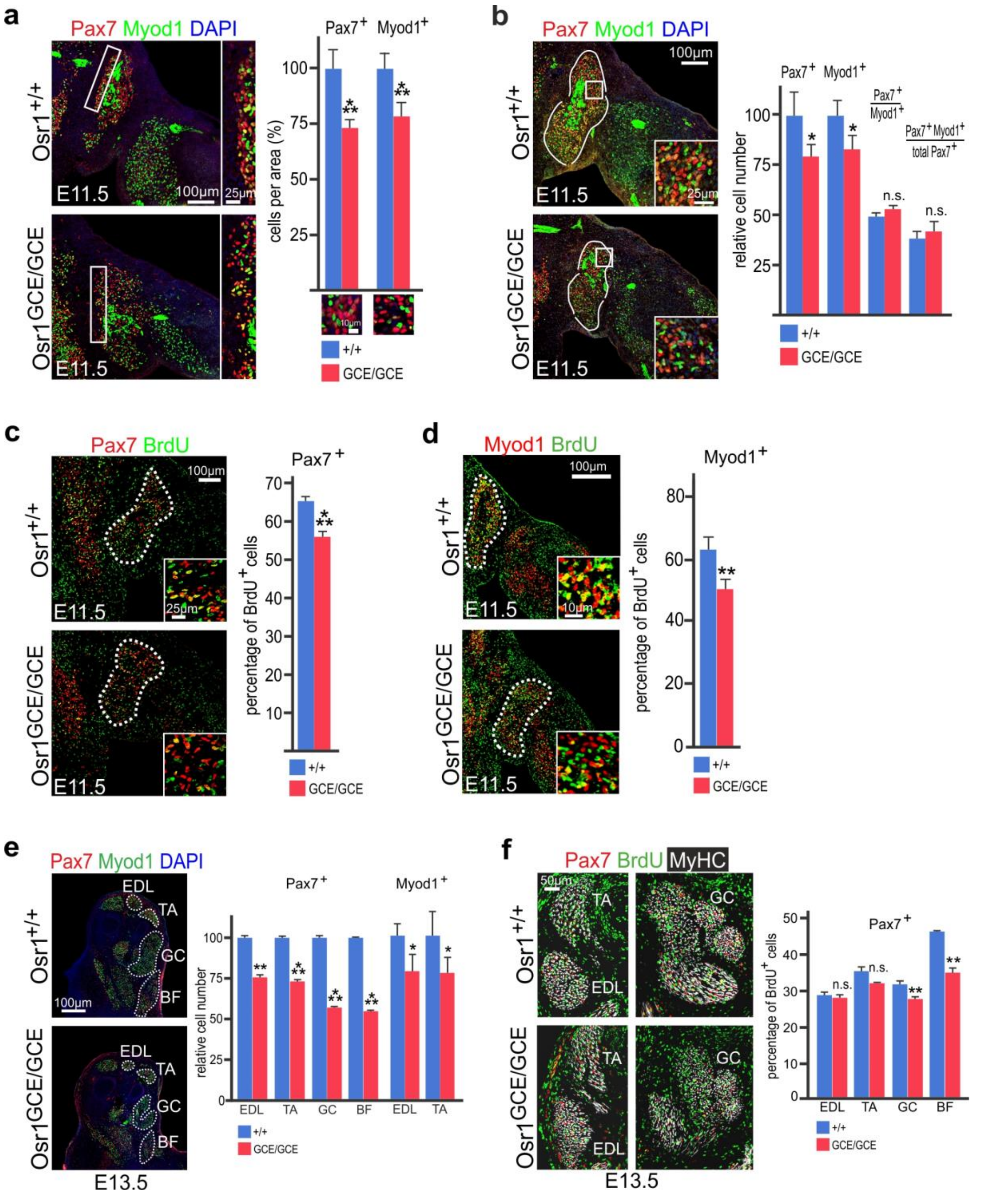

Supplementary Figure 8. Myogenesis defect in *Osr1*$^{GCE/GCE}$ embryonic and persistence of phenotype. (**a**) Immunolabeling for myogenic markers Pax7 and Myod1 on E11.5 limb sections shows proximal limb muscle primordia displaying low density of myogenic cells and fuzzy border of the muscle primordium of *Osr1* mutants (magnification of boxed region). Counting in defined areas within the muscle primordium revealed lower numbers of myogenic cells per area (n=3). (**b**) Reduced numbers of Pax7$^+$ and Myod1$^+$ cells in the proximal muscle primordium of *Osr1* mutants. The ratios of Pax7$^+$/Myod1$^+$ or Pax7$^+$Myod1$^+$/Pax7$^+$ cells is unaltered (n=3). (**c**) Pax7$^+$ and (**d**) Myod1$^+$ cell proliferation was assessed via BrdU labelling (Pax7: wt 65,8±1,0%, mut 56,9±1,1%; Myod1: wt 62,5±4,6%, mut 52,2±3,7%) (n=3). (**e**) Reduction of myogenic progenitor numbers at E13.5 in indicated muscles. EDL: Extensor digitorum longus; TA: Tibialis anterior; GC: Gastrocnemius; BF: Biceps femoris (n=3). (**f**) The rate of Pax7$^+$ progenitor proliferation is still reduced at E13.5 in several muscles of *Osr1*$^{GCE/GCE}$ embryos as compared to *Osr1*$^{+/+}$ controls (n=3). Error bars represent s.e.m. T-test: * = p<0.05; ** = p<0.01; *** = p<0.001.

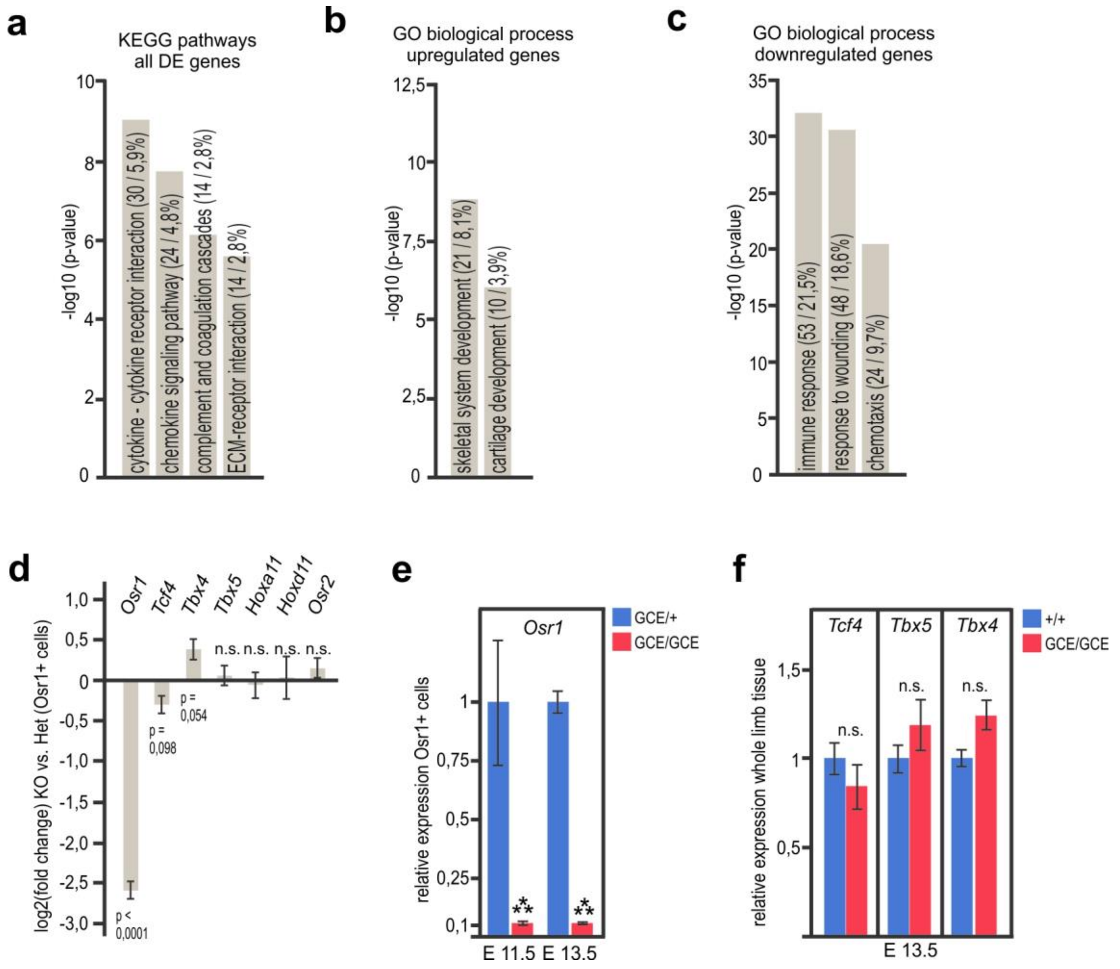

Supplementary Figure 9. Additional GO and KEGG analyses of *Osr1*$^{GCE/+}$ vs. *Osr1*$^{GCE/GCE}$ cells. RNA-Sequencing data and quantification of connective tissue-expressed transcription factors involved in muscle patterning. (**a**) KEGG pathway analysis on all DE genes. (**b, c**) GO analysis for "biological processes" from all upregulated or downregulated genes, respectively. (**d**) Depiction of RNA-Seq data shows no deregulation of *Tbx5, Hoxa11, Hoxd11* in FACS isolated Osr1^{+} cells (het vs. KO); *Tcf4* is mildly downregulated (relative expression 0,813, below cut-off) and *Tbx4* shows a significant mild upregulation (relative expression 1,304) in FACS isolated Osr1^{+} cells (het vs. KO). The *Osr1* paralog *Osr2* was not altered indicating no compensatory upregulation. (**e**) RT-qPCR quantification of *Osr1* expression in FACS isolated *Osr1*$^{GCE/+}$ vs. *Osr1*$^{GCE/GCE}$ cells (n=4). (**f**) RT-qPCR quantification of *Tcf4*, *Tbx4* and *Tbx5* in whole limb extracts shows no deregulation in *Osr1*$^{GCE/GCE}$ limbs as compared to wild type limbs from *Osr1*$^{+/+}$ littermates (n=3). Whole limb extracts were chosen over purified Osr1^{+} cells since Osr1^{+} cells do not necessarily overlap with limb Tcf4^{+}, Tbx4^{+} or Tbx5^{+} cells. Error bars represent s.e.m. T-test: *** = p<0.001.

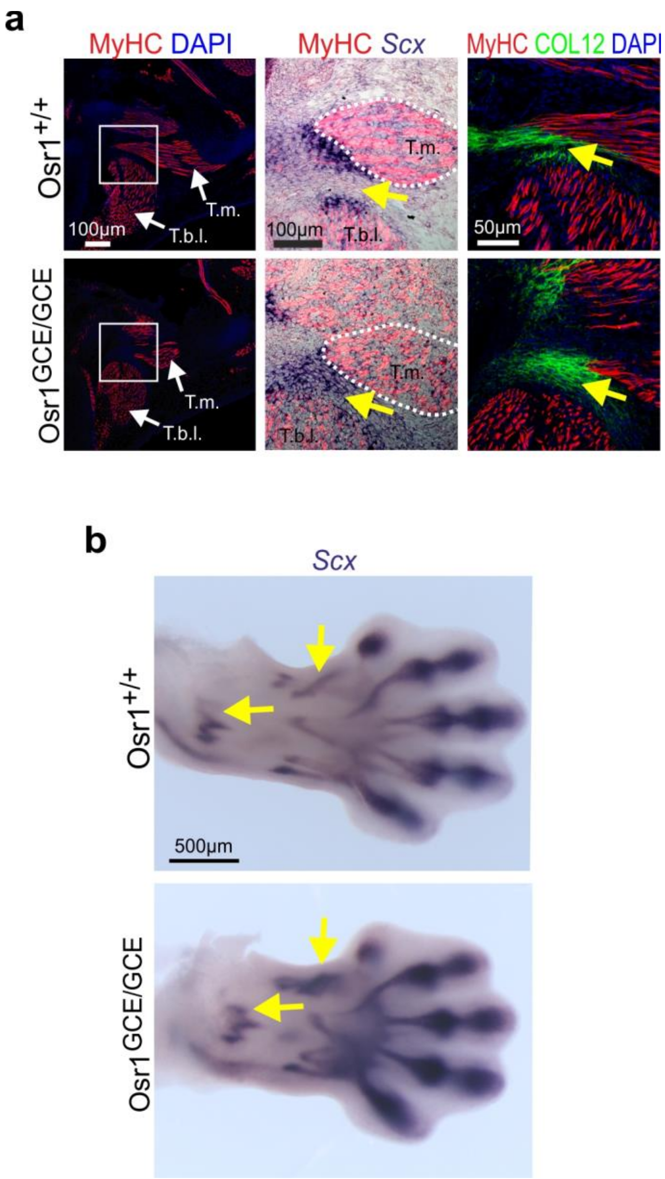

Supplementary Figure 10. Upregulation and misexpression of Scx. (**a**) Section *in situ*-hybridization for *Scx* and immunolabelling for the tendon-specific COL12 on adjacent sections show ectopic tendon cells embedded in a COL12 positive matrix between the Teres major (T.m.) and Triceps brachii lateralis (T.b.l.) muscles in *Osr1* mutants compared to wild-type mice, where the tendons appeared well separated. (**b**) Whole-mount ISH for Scx performed on E13.5 mouse embryos, dorsal sides of forelimbs are shown. Increased signal intensity and expanded expression is visible in Osr1$^{GCE/GCE}$ forelimbs (arrows).

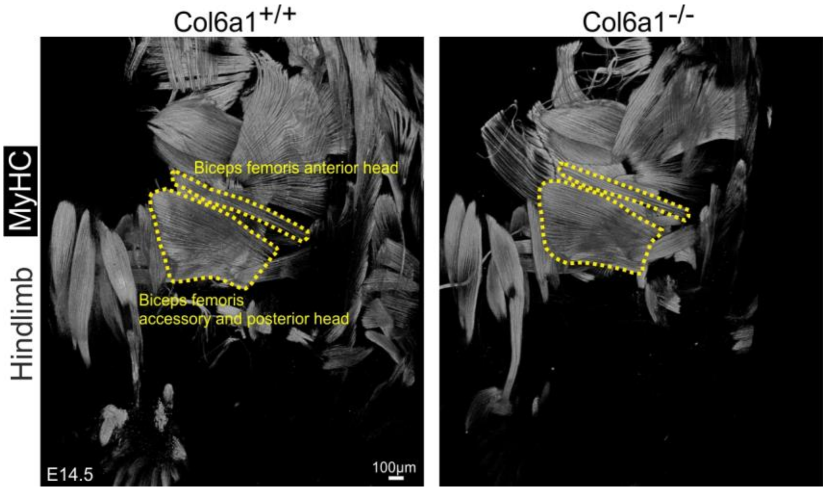

Supplementary Figure 11. *Col6a1* deficient embryos do not show muscle patterning defects. Images show 3D reconstructions of Myosin heavy chain (MyHc) immunolabelled E14.5 hindlimbs from *Col6a1*$^{+/+}$ and *Col6a1*$^{-/-}$ embryos. Dashed lines highlight the Biceps femoris anterior head and the Biceps femoris accessory and posterior head, muscles affected in *Osr1* and *Cxcr4* mutants. No significant changes in size and shape are visible in *Col6a1* mutants.

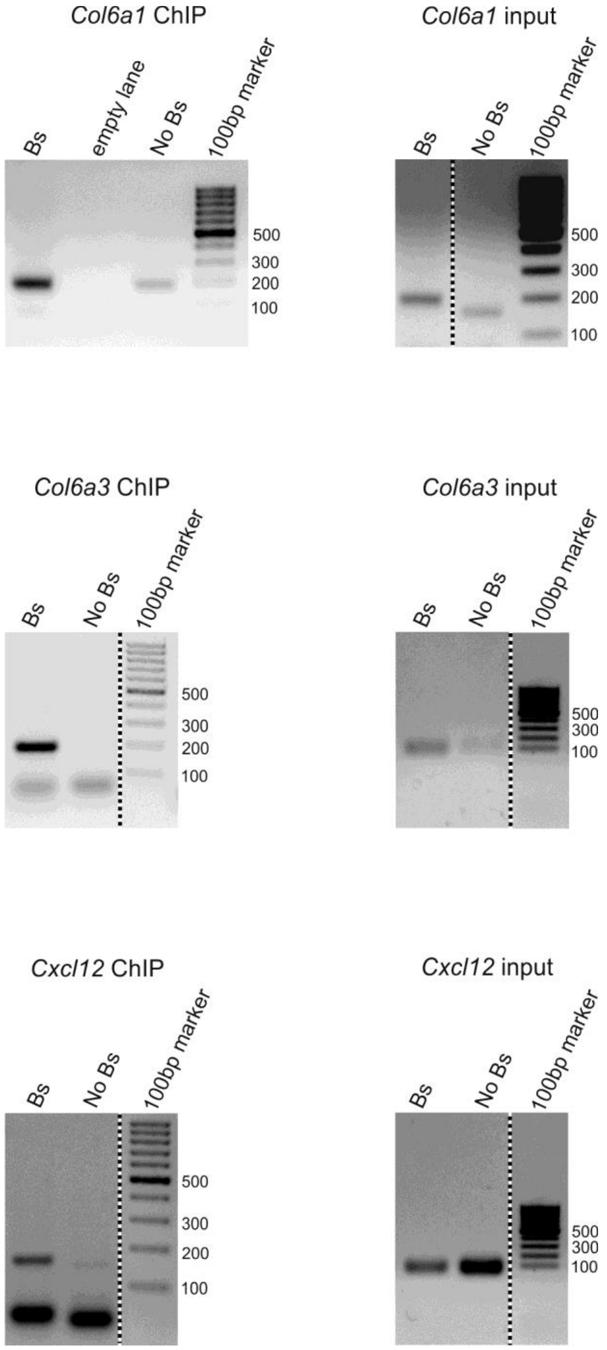

Supplementary Figure 12. Uncropped Gel electrophoresis lanes from ChIP experiments. Bs: putative Osr1 binding site; No Bs: comtrol region wiothout Osr1 binding motif. Size marker: 100 base pair ladder; band sizes are indicated in base pairs.

Supplementary Table 1 Expression levels of ECM genes in Osr1^{+} cells isolated from E13.5 *Osr1$^{GCE/+}$* embryo limbs

Gene	TPM rank	Mean TPM
Col3a1	6	4304
Col1a2	26	2607
Col1a1	67	1864
Sparc	86	1483
Postn	107	1227
Mfap2	178	721
Col6a1	180	713
Fn1	181	708
Col6a2	188	678
Col5a2	212	580
Mfap4	213	578
Dcn	232	550
Lum	231	548
Col5a1	276	465
Sulf2	349	386
Egfl6	446	322
Mmp14	452	317
Timp2	499	289
Col6a3	518	280
Islr	526	275
Bgn	546	268
Tgfbi	556	264
Lamb1	564	262
Ogn	572	260
Loxl2	577	255
Sepp1	623	241
Col2a1	639	236
Fbn2	657	231
Fbln1	709	255
Sulf1	748	208
Nid2	814	197
Adamts1	824	195
Nid1	855	188
Eln	901	181
Lox	917	178
Col4a1	970	172
Vcan	976	170
Emilin1	1014	167
Leprel2	1166	152
Kera	1179	146
Colec12	1206	144
Mmp11	1207	144
Net1	1240	142
Lepre1	1248	141

Shown are the mean transcripts per million (TPM) abundances of the DESeq2 normalized fragment counts and the respective rank for each gene. Genes shown here were selected from the top 10% of genes considered to be expressed (TPM $\geq$ 2).

Supplementary Table 2 mRNA abundances of mesenchymal stem cell and FAP surface marker genes in Osr1$^+$ cells

Protein marker / Gene	TPM rank	Mean TPM
CD29 (*Itgb1*)	306	427
PDGFRα (*Pdgfrα*)	401	348
CD106 (*Vcam1*)	1163	148
CD146 (*Mcam*)	2233	88
CD166 (*Alcam*)	2591	77
CD34 (*Cd34*)	3043	67
CD90 (*Thy1*)	4216	48
CD73 (*Nt5e*)	4601	43
CD44 (*Cd44*)	6914	25
CD105 (*Eng*)	8361	16
Ly-6A/E / Sca1 (*Ly6a*)	12531	2

Shown are the mean transcripts per million (TPM) abundances of the DESeq2 normalised fragment counts and the respective rank for each gene.

Supplementary table 3 Primary antibodies

Antibody	Clone	Conjugate	Concentration/ Dilution	Source
**Mouse anti-MyHC**	Monoclonal	Unconjugated	1:500	Chemicon
**Rabbit anti-GFP**	Polyclonal	Unconjugated	1 µg ml^{-1}	Torrey Pines Biolabs
**Chicken anti-GFP**	Polyclonal	Unconjugated	10 µg ml^{-1}	Abcam
**Goat anti-Desmin**	Polyclonal	Unconjugated	0.2 µg ml^{-1}	R&D Systems
**Guinea pig anti-Lbx1**	Polyclonal	Unconjugated	1:20.000	C. Birchmeier
**Mouse anti-Pax7**	Polyclonal	Unconjugated	1:50	DSHB
**Gunea pig anti-Pax7**	Polyclonal	Unconjugated	1:100	C. Birchmeier
**Goat anti-collagen IV**	Polyclonal	Unconjugated	10 µg ml^{-1}	Chemicon
**Rabbit anti-collagen VI**	Polyclonal	Unconjugated	1 µg ml^{-1}	Abcam
**Rabbit anti-α-SMA**	Polyclonal	Unconjugated	2 µg ml^{-1}	Abcam
**Hamster anti-Pecam1**	Polyclonal	Unconjugated	1:250	DSHB
**Rabbit anti-Laminin**	Polyclonal	Unconjugated	5 µg ml^{-1}	Sigma-Aldrich
**Rabbit anti-Col12a1**	Polyclonal	Unconjugated	1:500	M. Koch
**Rabbit anti-Col14a1**	Polyclonal	Unconjugated	1:500	M. Koch
**Mouse anti-Vinculin**	Monoclonal	Unconjugated	2 µg ml^{-1}	Santa Cruz
**Rabbit anti-NCadherin**	Polyclonal	Unconjugated	2 µg ml^{-1}	Santa Cruz
**Rabbit anti-TCF4**	Monoclonal	Unconjugated	0.2-2 µg ml^{-1}	Cell Signaling
**Goat anti-PDGFRα**	Polyclonal	Biotinylated	4 µg ml^{-1}	R&D Systems
**Rabbit anti-MyoD**	Polyclonal	Unconjugated	1:2500	C. Birchmeier
**Rabbit anti-MyoD**	Polyclonal	Unconjugated	2 µg ml^{-1}	Santa Cruz
**Rabbit anti-Vimentin**	Polyclonal	Unconjugated	1:100	Sanat Cruz
**Guinea pig anti-MyoD**	Polyclonal	Unconjugated	1:2.000	C. Birchmeier
**Mouse anti-Fibronectin**	Monoclonal	Unconjugated	1:100	Sigma-Aldrich
**Goat anti-C-Caspase3**	Polyclonal	Unconjugated	1:100	Cell Signaling
**Sheep anti-BrdU**	Polyclonal	Unconjugated	1:50	Abcam
**Mouse anti-Ki67**	Monoclonal	B56	1:100	BD Biosciences
**Rabbit anti-p44/p42 MAPK**	Polyclonal	Unconjugated	1:100	Cell Signaling
**Mouse anti-CyclinD1**	Monoclonal	Unconjugated	1:100	Santa Cruz
**Rabbit anti-FABP4**	Polyclonal	Unconjugated	1:300	Abcam
**Goat anti-collagen I**	Polyclonal	Unconjugated	1:100	SouthernBiotech
**Mouse anti-beta-Gal**	Monoclonal	Unconjugated	1:100	DSHB
**Rabbit anti-Myf5**	Polyclonal	Unconjugated	1:100	Santa Cruz
**Mouse anti-Myogenin**	Monoclonal	Unconjugated	1:200	Santa Cruz
**Mouse anti FLAG**	Monoclonal M2	Unconjugated	8µg ml^{-1}	Sigma Aldrich

Supplementary table 4 Secondary antibodies:

Antibody	Conjugate(s)	Source
**Donkey anti-mouse**	Alexa Fluor 488, 568 and 680	Molecular Probes
**Donkey anti-rabbit**	Alexa Fluor 488, 568 and 680	Molecular Probes
**Donkey anti-goat**	Alexa Fluor 488, 568 and 680	Molecular Probes
**Goat anti-hamster**	Alexa Fluor 488, 568	Molecular Probes
**Goat anti-guinea pig**	Alexa Fluor 488, 568	Molecular Probes
**Donkey anti-rat**	Alexa Fluor 488, 568	Molecular Probes
**Donkey anti-chicken**	Alexa Fluor 488, 568 and 680	Molecular Probes
**Donkey anti-sheep**	Alexa Fluor 488	Molecular Probes

Supplementary table 5 In-situ hybridisation probes

Gene	Reference
**Scx**	Schweitzer et al. 2001 [1]
**Osr1**	Stricker et al. 2006 [2]
**Myod1**	Sassoon et al. 1989 [3]
**Myogenin**	Sassoon et al. 1989 [3]

Supplementary table 6 FACS antibodies

**Hamster anti-CD29**	**HMb1-1**	**APC**	**1:100**	**eBioscience**
**Hamster anti-CD34**	HM31	PerCP-Cy5.5	1:100	BioLegend
**Rat anti-CD44**	IM7	PerCP-Cy5.5	1:100	eBioscience
**Rat anti-CD45**	30-F11	APC	1:100	BD Pharmigen
**Rat anti-CD73**	TY/11.8	PerCP-e710	1:100	eBioscience
**Mouse anti-CD90.1**	HIS51	PE	1:100	eBioscience
**Rat anti-CD105**	MJ7/18	APC	1:100	eBioscience
**Rat anti-CD140a**	APA5	PE	1:100	eBioscience
**Rat anti-CD166**	eBioALC48	APC	1:100	eBioscience
**Rat anti-T119**	TER-119	APC	1:100	BD Pharmigen
**Rat anti Ly-6A/E**	E13-161.7	PE/Cy7	1:100	BioLegend
**Anti rat α7-integrin**	R2F2	PE	1:1000	AbLab
**Anti mouse CD31**	390	APC	1:500	eBioscience
**Anti mouse Ly-6A/E (Sca-1)**	D7	APC/Cy7	1:1000	BioLegend
**Anti mouse CD34**	RAM34	eFluor® 450	1:500	eBioscience

Supplementary table 7 Primer sequences (RT-qPCR):

Gene	Forward	Reverse
Gadph	CTGCACCACCAACTGCTTAG	GGATGCAGGGATGATGTTCT
Col6a1	CGTGGATGCGGTCAAGTA	CCAGGTGTTTGGCCTCATTT
Col6a2	TTCCCTGCCAAACAGAGC	ATATTGCAACAGAGCCATGC
Col6a3	AGGCCGTACTCAAGCTTTCC	AGCAAACATGGCAGGTAAGG
Osr1	GCACACTGATGAGCGACCT	TGTAGCGTCTTGTGGACAGC
Osr2	CACACAGACGAGAGGCCATA	GCAGCTGTAGGGCTTGATGT
Tbx4	CCAACTCAGAGGGACTCCA	TCAGCATCTGCTGGTCGTA
Tbx5	AGCTCTCTCCACCTCATCCA	CCGAGCGATAGAAGGTGTC
Cxcl12	GCTCCACCCACAAGGTTAAG	CTGGCAGAAGGCCTTGAATA
Col3a1	CTAAAATTCTGCCACCCCGAA	AGGATCAACCCAGTATTCTCCACTC
Tcf4	AAGCCTCCAGAGCAGACAAA	TAAGTGCGGAGGTGGATTTC
Scx	CCTTCTGCCTCAGCAACCAG	GGTCCAAAGTGGGGCTCTCCGTGACT
Tnmd	AACACTTCTGGCCCGAGGTAT	AAGTGTGCTCCATGTCATAGGTTTT
Mkx	AGTAAAGACAGTCAAGCTGCCACTG	TCCTGGCCACTCTAGAAGCG
Runx2	GGTCCCCGGGAACCAA	GGCGATCAGAGAACAAACTAGGTTT
Sox5	GCTCCACCCACAAGGTTAAG	CTGGCAGAAGGCCTTGAATA
Sox9	GCTCCACCCACAAGGTTAAG	CTGGCAGAAGGCCTTGAATA
Actb	CTGTATTCCCCTCCATCGTG	GGAGAGCATAGCCCTCGTAG
Bmp4	GGATCTTTACCGGCTCCAG	GCTGCTGAGGTTGAAGAGGA
Col5a3	CCGGAGACTGGATCAGCTT	GGTCACCCTGTGGAATCCT
Lum	TGCTCGAGCTTGATCTCTCC	AAGCGCAGATGCTTGATCTT
Dcn	ACCCTGACAATCCCCTGATA	TTCTTGAAGGCCCCTTCTTT
Nid2	GGGCCTTTGCTTTGTACAGT	TGCACTCGCAGGTGTAGTCT

Supplementary table 8 Primer sequences (ChIP-PCR):

Gene		Forward	Reverse
**Col6a3**	Binding site 1	TTAGCCCCATGGTTCTTGAC	TTTTATGGCCTCTGGCAGTC
	Binding site 2	ACATCCCAAGAGGACAGGTG	TGATCTCGCTTTCTGCACAT
	Control 1/2 (no Bs)	AAGCCAAGCAGAGAGCAGAG	TGGGCTTTCACATCACTTCA
**Col6a1**	Binding site 1	TACCAGCTGAGCCATCTCAC	CGACTCTAGGAGTGCATGCTT
	Binding site 2	TGTTAGCCCCATCACTGTCA	TGTTGGACCCATGCAGACTA
	Control 1/2 (no Bs)	TCCACCTTTGTTTCTTTGTGG	GGAAACAGGAGGAGATTGAGG
**Lum**	Binding site 1	AGGGGCTTCCAAGCTAAAAG	GGAGGTTGCAAGTTTAAACCAA
	Binding site 2	TACCAGCCTTTAGGGCTTTG	TCTCAACAAGCATGCAATCA
	Control 1/2 (no Bs)	TTAGGGACTTGGGGGAGAGT	GCCCGCTTGTATTTGTGATT
**Cxcl12**	Binding site 1	AAGCAATACTTTGGTCAAAGAGAAA	CCAGCCCAGAATTCTTCATT
	Control 1 (no Bs)	GGAGGCACTGTGGTATTTGG	GGCCCAAAGGAGTAAATCAA
	Binding site 2	CAGCAATAGGAACAACAATAACAA	TGGTCTCCTTCCATTGGTTT
	Binding site 3	CACCAACGCATGCTGTAAGA	AAACCCTCTGTGGCTGTGAG
	Control 2/3 (no Bs)	CACATTGCAGTGGATTTTGG	CTTTCCATGTGACTGCTGGA

Blue: Putative Osr1 binding sites that were positive in ChIP, and respective control sites without binding motif.

Supplementary References

1. Schweitzer R, *et al.* Analysis of the tendon cell fate using Scleraxis, a specific marker for tendons and ligaments. *Development* **128**, 3855-3866 (2001).

2. Stricker S, Brieske N, Haupt J, Mundlos S. Comparative expression pattern of Odd-skipped related genes Osr1 and Osr2 in chick embryonic development. *Gene Expr Patterns* **6**, 826-834 (2006).

3. Sassoon D, *et al.* Expression of two myogenic regulatory factors myogenin and MyoD1 during mouse embryogenesis. *Nature* **341**, 303-307 (1989).
